# RANDOMIZED CONTROLLED TRIAL OF COGNITIVE BEHAVIOURAL THERAPY VERSUS HEALTH EDUCATION FOR SLEEP DISTURBANCE AND FATIGUE FOLLOWING STROKE AND TRAUMATIC BRAIN INJURY

**DOI:** 10.2340/jrm.v57.41302

**Published:** 2025-01-03

**Authors:** Lucy YMER, Adam MCKAY, Dana WONG, Kate FRENCHAM, Natalie GRIMA, Monique ROPER, Sylvia NGUYEN, Jade MURRAY, Gershon SPITZ, Jennie PONSFORD

**Affiliations:** 1 Monash Epworth Rehabilitation Research Centre, School of Psychological Sciences, Monash University, Melbourne, VIC; 2 Epworth Healthcare, Melbourne, VIC; 3 School of Psychology and Public Health, La Trobe University, Melbourne, VIC, Australia

**Keywords:** acquired brain injury, stroke, sleep, fatigue, cognitive behavioural therapy, health education

## Abstract

**Objective:**

Evaluate efficacy of cognitive behavioural therapy for sleep and fatigue adapted for brain injury relative to health education control in alleviating sleep disturbance and fatigue after acquired brain injury.

**Design:**

Parallel groups randomized controlled trial.

**Subjects:**

126 community dwelling adults with stroke or traumatic brain injury.

**Methods:**

Participants were randomized 2:1 to receive 8-weeks of cognitive behavioural therapy for sleep and fatigue (*n* = 86) or health education (*n* = 40). The Pittsburgh Sleep Quality Index was assessed pre- and post-treatment, and 2 and 4-months post-treatment, with secondary measures of insomnia, fatigue, sleepiness, mood, quality of life, activity levels, self-efficacy, and actigraphy.

**Results:**

Both groups showed improved sleep by 4-month follow-up. However, cognitive behavioural therapy for sleep and fatigue had significantly larger and more rapid improvements than health education immediately post-treatment (*β* = –1.50, *p* < 0.001, 95% confidence interval –2.35 to –0.64). There were no significant between-groups differences in fatigue; however, cognitive behavioural therapy for sleep and fatigue showed within-group gains on both fatigue measures immediately post-treatment and over time (*β* = –0.29, *p* = 0.047, 95% confidence interval –0.58 to –0.01). Health education had delayed improvements at 4-month follow-up on 1 fatigue measure.

**Conclusions:**

Both cognitive behavioural therapy for sleep and fatigue and health education led to improvement in sleep and fatigue; however, effects were larger and more rapid for cognitive behavioural therapy for sleep and fatigue immediately post-treatment. This supports the efficacy of cognitive behavioural therapy for sleep and fatigue in acquired brain injury but also highlights that health education may result in delayed improvements in symptoms.

*ANZCTR Trial registration numbers*: 1261700087830; 12617000879369

## LAY ABSTRACT

Sleep and fatigue problems are common and disabling for people with brain injury and can impact multiple areas of daily life and physical functioning. This study compared a combined cognitive behavioural therapy treatment for sleep and fatigue with a health education intervention. In total, 126 adults with a traumatic brain injury or stroke took part in the study and completed 8 weekly one-on-one sessions of either cognitive behavioural therapy for sleep and fatigue or health education with a neuropsychologist. They completed questionnaires before treatment, after treatment and at 2 months and 4 months after treatment. These measures assessed sleep, fatigue, mood, quality of life, self-efficacy, and activity levels. Overall, both groups showed improvements in sleep and fatigue symptoms, but those who received cognitive behavioural therapy for sleep and fatigue had larger and more rapid improvements than health education. Overall, these findings demonstrate the benefits of cognitive behavioural therapy for sleep and fatigue, but also highlight that health education can bring slow improvements in sleep and fatigue after brain injury.

Sleep disturbance and fatigue are prevalent and debilitating symptoms of acquired brain injury (ABI), impacting rehabilitation, activity participation, and quality of life (1, 2). Approximately 50% of individuals who sustain a traumatic brain injury (TBI) and 42% of stroke survivors report sleep disturbances (3, 4), and 48–70% report persistent fatigue (5, 6) over months or years after injury (7). These often, but do not always, co-occur. The causes of sleep disturbance and fatigue after ABI are multifactorial, including biological factors underpinned by injury to sleep–wake or arousal centres that disrupt homeostatic or circadian rhythms, cognitive impairments that make information processing more effortful, as well as depression, anxiety, and chronic pain (8–10). These symptoms significantly interfere with daily activities (1). It is therefore vital to treat these symptoms effectively to maximize participation and quality of life for people with ABI.

Despite the high prevalence and negative impact of sleep disturbance and fatigue on functional, physical, cognitive, and social outcomes after ABI, evidence of effective treatments is scarce. The co-occurrence of sleep disturbance and fatigue with psychological factors, such as depression and anxiety, is well established and these relationships are complex and thought to be bi-directional (8). As such, psychological approaches to treatment such as cognitive behavioural therapy (CBT) are warranted. However, therapy approaches need to be adapted to accommodate common cognitive impairments after ABI. Individuals who experience deficits in attention, learning, memory, executive functioning, and social communication may have difficulty understanding new or complex concepts presented in therapy, remembering materials from previous sessions, completing homework, implementing strategies, or absorbing large amounts of information presented in therapy. Moreover, geographical and financial barriers to accessing treatment and services are frequently exacerbated for individuals with brain injury, and therefore consideration and evaluation of treatment delivery via videoconferencing is important to enhance accessibility (11).

We developed an 8-week CBT intervention to treat sleep disturbance and fatigue (CBT-SF) after ABI, adapted to accommodate cognitive difficulties. Pilot studies have shown promising effects of CBT-SF on sleep quality and fatigue in individuals with TBI and stroke compared with treatment-as-usual (12, 13) and in comparison with an active health education (HE) control intervention (14, 15). In an internal pilot study Ymer et al. (15) found that delivery of intervention via videoconference did not appear to impact response to treatment. However, that pilot trial was underpowered. The current study therefore extends the internal pilot trial of Ymer et al. (15), reporting the results of the full randomized controlled trial comparing the efficacy of CBT-SF with HE in individuals with stroke and TBI, delivered in-person and via telehealth.

It was hypothesized that individuals receiving CBT-SF would report greater improvements in sleep quality than those receiving HE and maintain gains over time, and that on secondary outcomes, participants receiving CBT-SF would report greater improvements on measures of fatigue, depression, anxiety, health-related quality of life, self-efficacy, actigraphy variables (sleep efficiency and sleep onset latency), and on time spent in productive activity than those receiving HE.

## METHODS

### Design

A randomized parallel groups design was approved by the relevant hospital Human Research Ethics Committees (RES-19-0000178E). Prospective registration was undertaken with Australian New Zealand Clinical Trials Registry for TBI (12617000878370) and stroke (12617000879369) in June 2017. The pilot randomized controlled trial (RCT) was an internal pilot trial (15). No changes were made to measures or procedures and therefore those data are included in the current dataset, consistent with the procedures recommended by Bond et al. (16). The trial is reported in accordance with CONSORT guidelines.

Individuals participated face-to-face or via videoconferencing according to preference. Assessments were completed at baseline, post-treatment, 2 months post-treatment, and 4 months post-treatment. Researchers conducting follow-ups were blinded to treatment allocation. Therapists and participants could not be blinded but both interventions were presented as active interventions. Participants were blinded to hypotheses.

**Fig. 1:**
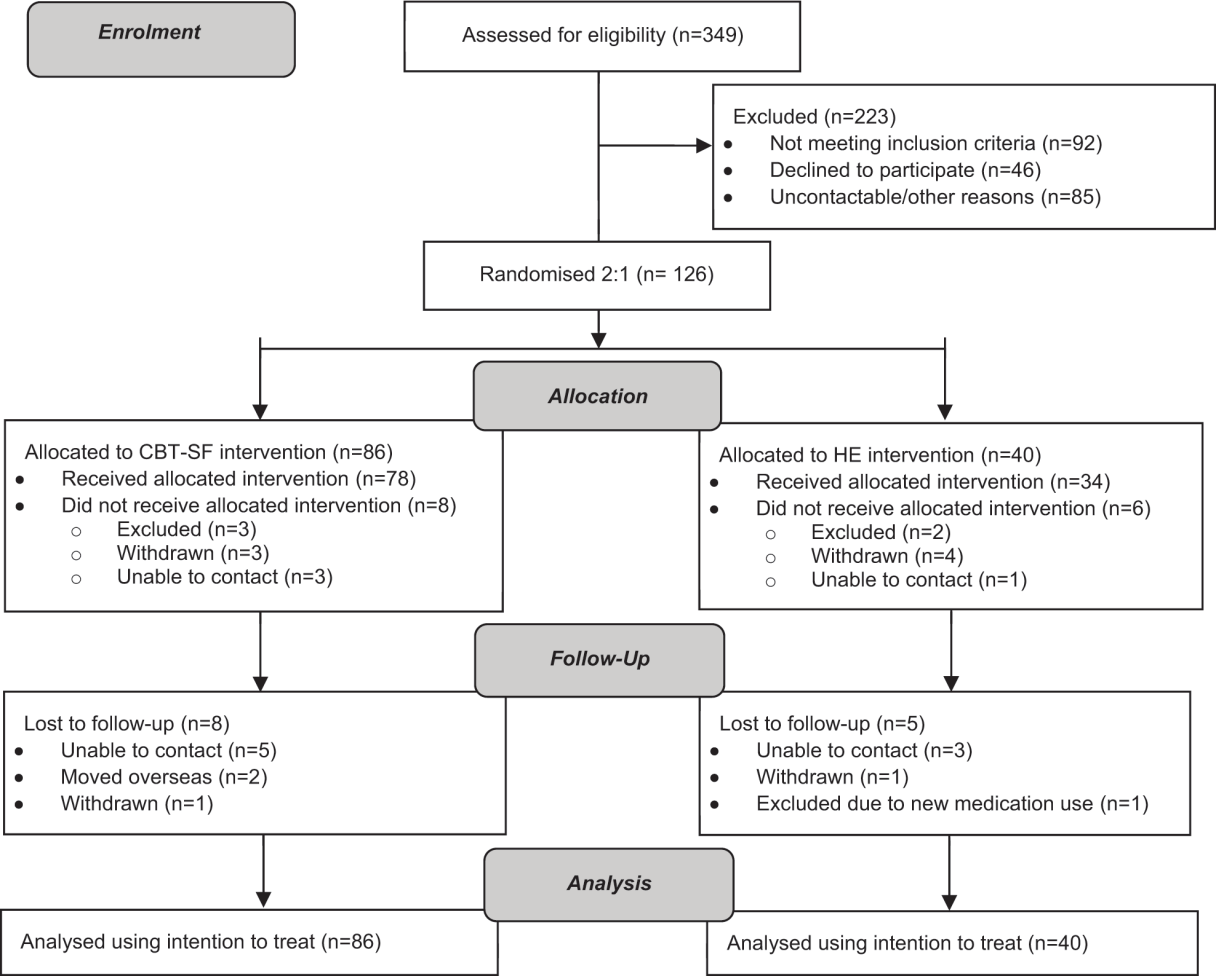
Flow of participants through each stage of the randomized controlled trial. CBT-SF: Cognitive Behavioural Therapy for Sleep Disturbance and Fatigue; HE: Health Education.

After providing informed consent participants completed a baseline assessment and were randomized to CBT-SF or HE (Fig. 1). Stratified randomization using Stata ralloc procedure (StataCorp LLC, College Station, TX, USA) was undertaken by an independent researcher, using a random length permuted blocks algorithm based on median baseline sleep quality. Given outcomes favouring CBT-SF in the pilot study compared with treatment-as-usual (12, 13), the current sample was randomized 2:1 into CBT-SF and HE to allow additional data for analysis of predictors of response to CBT-SF. Separate randomization schedules were generated for stroke and TBI, and further separated into face-to-face and telehealth modes. These were sealed and opened by the study coordinator after the baseline assessment.

### Participants

Individuals in Australia with a stroke or TBI, aged 16–71, were recruited via convenience sampling from a longitudinal TBI research database (17) and advertisements to ABI clinicians and stroke support groups/websites. Participants were followed up between June 2017 and October 2023.

Participants had a documented history of TBI or stroke at least 3 months prior to enrolment. Participants with TBI required a medically documented history of blunt head trauma with loss of consciousness, Glasgow Coma Scale 3–14, post-traumatic amnesia (PTA) and/or other neurological abnormalities/intracranial lesions. Participants with stroke had diagnostic evidence of stroke on neuroimaging. All participants had sleep disturbance and/or fatigue, rating > 5 on the Pittsburgh Sleep Quality Index (PSQI; 18), and/or ≥ 4 on the Fatigue Severity Scale (FSS; 19). Participants with aphasia were included provided they could adequately engage with study requirements. All participants had adequate English skills, cognitive ability, and visual acuity to complete the study, assessed during eligibility screening and baseline assessment by a neuropsychologist.

Exclusion criteria included “high-risk” scores on the Berlin Questionnaire for sleep apnoea (20) or untreated symptomatic sleep apnoea. Participants with another neurological disorder, pre-injury diagnosed sleep disorder, or chronic fatigue syndrome were excluded, as were participants requiring surgery, travelling across time zones, or engaging in nightshift work in the previous 6 weeks. Medications were allowed if maintained at stable dosage, excluding hypnotic medications or illicit drugs.

### Power analysis

The internal pilot trial showed recruitment and randomization methods and the HE intervention were feasible to implement. As no methodological changes were required, we proceeded to the main RCT and retained participant data, as recommended by Bond et al. (16). Power analysis based on Ymer et al. (15) found a statistically significant group difference on the PSQI comparing CBT and HE, *t*(49) = 1.56, *p* < 0.001, with a Cohen’s *d* effect size of 0.88. Sample size analysis was based on an independent samples test, using Stata 18 (21), with 80% power. Due to the lower numbers in HE, a conservative effect size of 0.75 was adopted, and a 2-tailed alpha of 0.01, allowing for 20% attrition. Group sizes of 66 CBT and 33 HE participants were estimated, with a total of 125 allowing for attrition.

### Measures

Demographics, injury data, medication, and health service engagement were obtained via interview and medical record review with participant consent. Measures taken for planned secondary analyses included pre-morbid intellectual function using the National Adult Reading Test (NART; 22), and verbal learning and memory assessed via the California Verbal Learning Test–2nd edition (CVLT-II; 23). Psychiatric screening and baseline pain were assessed using the Health of the Nation Outcome Scale–Acquired Brain Injury (HoNOS-ABI; 24) and Brief Pain Inventory (BPI; 25).

### Primary outcome

The PSQI has 19-items assessing subjective sleep quality over the past month, with global scores ranging from 0–21 (18). Higher scores indicate poorer sleep quality, with a clinical cut-off of > 5 and a clinically meaningful change of ≥ 3 (18). In TBI, the PSQI has 93% sensitivity and 100% specificity for identifying insomnia (26), and shows strong reliability, moderate structural validity, and good internal consistency (27).

### Secondary outcomes

The Insomnia Severity Index (ISI) assessed insomnia symptomatology, with lower scores indicating less insomnia across 7 items on a 5-point scale (28). The ISI has excellent internal consistency and strong convergent and divergent validity in clinical samples and TBI (28, 29). The impact of fatigue on daily living was measured using FSS, averaging 9 items on a 7-point scale, with ≥ 4 indicating clinically significant fatigue (19). In stroke samples the FSS has excellent internal consistency, good test–retest reliability, and moderate correlations with other fatigue measures (30). Fatigue severity and impact over the previous 24 h was assessed by the Brief Fatigue Inventory (BFI), with nine items rated 0–10 (31). The BFI has good reliability and validity for cancer-related fatigue (32), and was sensitive to change in our pilot trial (13). Excessive daytime sleepiness was evaluated using the Epworth Sleepiness Scale (ESS), rating likelihood of falling asleep in certain situations on a 4-point scale (33). The ESS has good concurrent validity with the PSQI (26).

The Short Form Health Survey version 2 (SF36v2) measured quality of life, via the Mental Component Summary (MCS) and Physical Component Summary (PCS; 34). Depression and anxiety symptomatology were assessed with the Hospital Anxiety and Depression Scale (HADS-D, HADS-A; 35). Confidence in managing symptoms of ABI was evaluated using the Self-efficacy for Managing Brain Injury questionnaire, adapted from the Stanford Self-Efficacy for managing Chronic Disease 6-Item Scale (36). A 1-week activity diary recorded daily physical and mental activity, rest and sleep, converted into a percentage of time spent in productive activity.

### Objective sleep measurement

Sleep efficiency and sleep onset latency (SOL) data were obtained using wrist actigraphy devices (Actiwatch 2 and Actiwatch Spectrum Plus, Phillips Respironics, Bend, OR, USA) over 2 weeks at each time point. Self-reported sleep diaries (bed and wake times) were utilized to adjust rest intervals to enhance accuracy of estimated sleep times. Data were excluded where < 5/14 nights were available.

### Interventions

Trained and experienced clinical neuropsychologists with expertise in ABI and CBT administered both treatments, via face-to-face and telehealth (using videoconference). Therapy content across in-person and telehealth modes was identical, with therapy materials including handouts mailed or emailed to telehealth participants.

### Cognitive behavioural therapy

CBT-SF (12, 13) included 7 treatment modules delivered across 8 x 1-hour individual sessions, addressing maladaptive beliefs and behaviours perpetuating sleep and fatigue symptoms (Table I). While there was a requirement for participants to complete all modules, therapy content was tailored to participants’ presenting complaints and individualized goals. Each treatment module incorporated core CBT principles. Sleep modules encompassed sleep hygiene, stimulus control, sleep restriction, and relaxation/stress training. Fatigue management techniques encouraged flexible pacing techniques via activity scheduling, task analysis, and compensatory strategies. Cognitive therapy techniques centred on thought restructuring, and the final session explored relapse prevention. Homework was assigned and reviewed weekly. CBT-SF included adaptations for common cognitive impairments after ABI, by using increased session structure, concrete concepts, regular repetition, explicit discussion as to how homework and strategies would be implemented, use of reminders, written handouts, and additional support from therapists to identify unhelpful thought patterns or behaviours.

**Table I:** Summary of session content for each intervention

**Cognitive Behavio**u**ral Therapy for Sleep Disturbance and Fatigue (CBT-SF)**	
**Module**	Session content
**1**	Rationale for CBT-SF and psychoeducation about post-ABI sleep and fatigue Goal setting and strategies to build motivation if necessary Self-monitoring of daily activities and subjective fatigue levels
**2**	Revision of self-monitoring diary Planning and setting an initial activity programme including regular sleep/wake times
**3**	Revision of activity programme, with modifications made as needed Sleep education and sleep hygiene principles
**4**	Revision of activity programme, with gradual increases/decreases in activity levels Use of cognitive therapy to address maladaptive thoughts and behaviour patterns
**5**	Revision of activity programme, exercise, and sleep hygiene practices Implementation of tailored sleep interventions
**6**	Revision of activity programme, exercise, and sleep intervention Learning practical strategies to minimize physical and mental fatigue
**7**	Revision of activity programme, exercise, sleep intervention, and fatigue strategies Relapse prevention
**Health Education**	
**Module**	Session content
**1**	Introduction to the intervention and general TBI/stroke education Common cognitive, behavioural, and emotional sequalae following ABI
**2**	Normal sleep and disturbed sleep following ABI: types, frequency, and causes Impact of sleep disturbance on everyday life
**3**	Definition and types of fatigue, causes, and associated factors Impact of fatigue on everyday life
**4**	Types of exercise and benefits, relationship between exercise, sleep, and fatigue How stress affects mind and body, relationship between stress, sleep, and fatigue
**5**	Healthy diet and brain health, relationship between diet, sleep, and fatigue Impact of alcohol and substance use on the brain, and impacts on sleep and fatigue
**6**	Common cognitive difficulties following ABI Relationship between cognitive difficulties, sleep, and fatigue
**7**	Importance of recovery and rehabilitation after ABI Emotional stages of recovery and factors affecting recovery

ABI: acquired brain injury; TBI: traumatic brain injury.

### Health education

The HE intervention (Table I) was developed for the present study and tailored to an ABI population (14). It was structured identically to CBT-SF, incorporating 7 modules across 8 x 1-hour sessions. It was designed to account for non-specific therapy effects including therapist contact, therapist empathy, therapeutic alliance, and treatment expectations (37). HE provided information and non-individualized strategies for health topics associated with ABI, sleep, and fatigue, avoiding components or therapeutic processes of CBT-SF. There were no individualized goals, tailored strategies, or specific homework tasks. Adaptations for cognitive impairments included breakdown and simplification of information, repetition of key points, and take-home handouts and worksheets.

### Treatment integrity

An independent psychologist with expertise in CBT, not part of the research team, rated 1–2 randomly selected audio recordings for each participant, from 1 (unacceptable) to 8 (excellent) for: (*i*) overall session delivery; (*ii*) adherence to module being delivered; and (*iii*) competency in module delivery. For the CBT-SF group, “overall session delivery” was defined as adherence to a general CBT approach, including setting an agenda, presenting a rationale for therapeutic tasks, reviewing or assigning homework, and maintaining the therapeutic relationship. In HE, “overall delivery of the session” encompassed initial discussion of the participant’s symptoms, presentation of educational information, discussion of the participants’ experiences of the specific topic, and no use of individualized strategies. Adherence and competency in delivering the modules were rated specific to the module being delivered, using component guidelines within the treatment manuals. Therapists also received regular group and individual supervision.

### Data analysis

Data were analysed using Jamovi 2.3 (38) and RStudio 2021.09.0 (39) following intention-to-treat principles. Following assumption checking, baseline variables were compared between groups using independent samples *t*-tests and χ^2^ tests. Changes in CBT-SF and HE groups over time were analysed using linear mixed-effect regression models, with time, treatment, and group-by-time interaction as fixed effects, and random effects for participant intercepts. Covariates of injury type, delivery mode, and variables on which groups differed at baseline were assessed and included in analysis if significantly impacting analysis outcome. Simple effects were explored even in the absence of a significant omnibus effect, using *a priori* simple contrasts for between-group and within-group differences over time, with Bonferroni corrections. Participants with clinically meaningful change on the PSQI compared with baseline were identified using a cutoff of 3 (18). Secondary analyses examined regression models on the PSQI and FSS within TBI and stroke groups separately to establish any substantial differences between outcomes for different injury types.

## RESULTS

Participants were 126 individuals, 51 (40%) with a TBI and 75 (60%) stroke survivors (Table II; Tables SI and SII). Approximately 76% of participants were enrolled via telehealth, a higher number than anticipated due to COVID-19 restrictions and distant location. Approximately 86% of participants had both clinically significant sleep disturbance and fatigue at baseline. There were no significant differences between groups at baseline for demographic, injury, or cognitive variables, although participants in the CBT group had significantly higher pain and anxiety than HE. Five participants were in the impaired range for verbal learning trials on the CVLT-II, and 5 participants had aphasia.

**Table II:** Demographic and injury characteristics by treatment condition at baseline

	CBT-SF (*n* = 86)	Health education (*n* = 40)	Range	*p*-value
Demographics				
Age at study entry, mean (SD)	47.06 (14.62)	49.47 (13.75)	19–71	0.382
Sex (% male)	53%	50%	–	0.715
Years of education, mean (SD)	13.57 (1.87)	13.46 (2.32)§	9–18	0.782
Mode (% telehealth)	76%	78%	–	0.813
Injury characteristics				
Injury type (% traumatic brain injury)	42%	38%	–	0.643
Time since injury (months), mean (SD)	52.74 (56.77)§	63.64 (71.31)§	5–287	0.363
Injury severity (for traumatic brain injury), %			–	0.638
Mild	17%	8%		
Moderate	28%	38%		
Severe	55%	54%		
Post-traumatic amnesia in days (for traumatic brain injury), mean (SD)	19.75 (25.23)†	12.00 (12.06)†	< 1–109	0.409
Glasgow Coma Scale score (for traumatic brain injury), mean (SD)	9.33 (4.78)†	9.18 (5.02)†	3–15	0.930
Stroke mechanism (% ischaemic)	69%‡	71%§	–	0.856
Stroke hemisphere (%)				0.216
Right	40%‡	54%§	–	
Left	41%‡	21%§		
Bilateral	19%‡	25%§		
Cognitive characteristics				
National Adult Reading Test Intelligence Quotient, mean (SD)	110.46 (6.57)†	111.68 (7.32)‡	89–121	0.369
CVLT-II T-score (trials 1–5), mean (SD)	48.45 (12.52)‡	47.61 (12.25)‡	12–77	0.728
Psychological measures, mean (SD)				
Health of the Nation Outcome Scale–Acquired Brain Injury score	7.31 (4.06)	6.89 (4.30)‡	0–19	0.603
Baseline Hospital Anxiety and Depression Scale–Depression subscale	6.56 (3.54)	6.05 (3.72)‡	0–17	0.472
Baseline Hospital Anxiety and Depression Scale–Anxiety subscale	8.41 (4.08)	6.55 (3.94)‡	0–20	0.020
Baseline Brief Pain Inventory	4.20 (1.62)	3.12 (1.57)	0–7	0.039

CBT-SF: cognitive behavioural therapy for sleep disturbance and fatigue; CVLT-II: California Verbal Learning Test–second edition; SD: standard deviation.

† Missing data from 4–8 participants.

‡ Missing data from 2 participants.

§ Missing data from 1 participant.

### Treatment integrity

Ratings for all criteria were “Very High” to “Excellent” on average, across both groups (range 6–8).

### Efficacy analysis

**Fig. 2:**
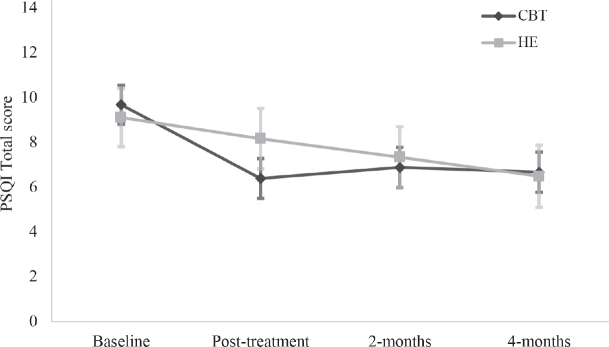
Estimated marginal means with standard error bars on the Pittsburgh Sleep Quality Index (PSQI) for each group at each time point.

**Fig. 3:**
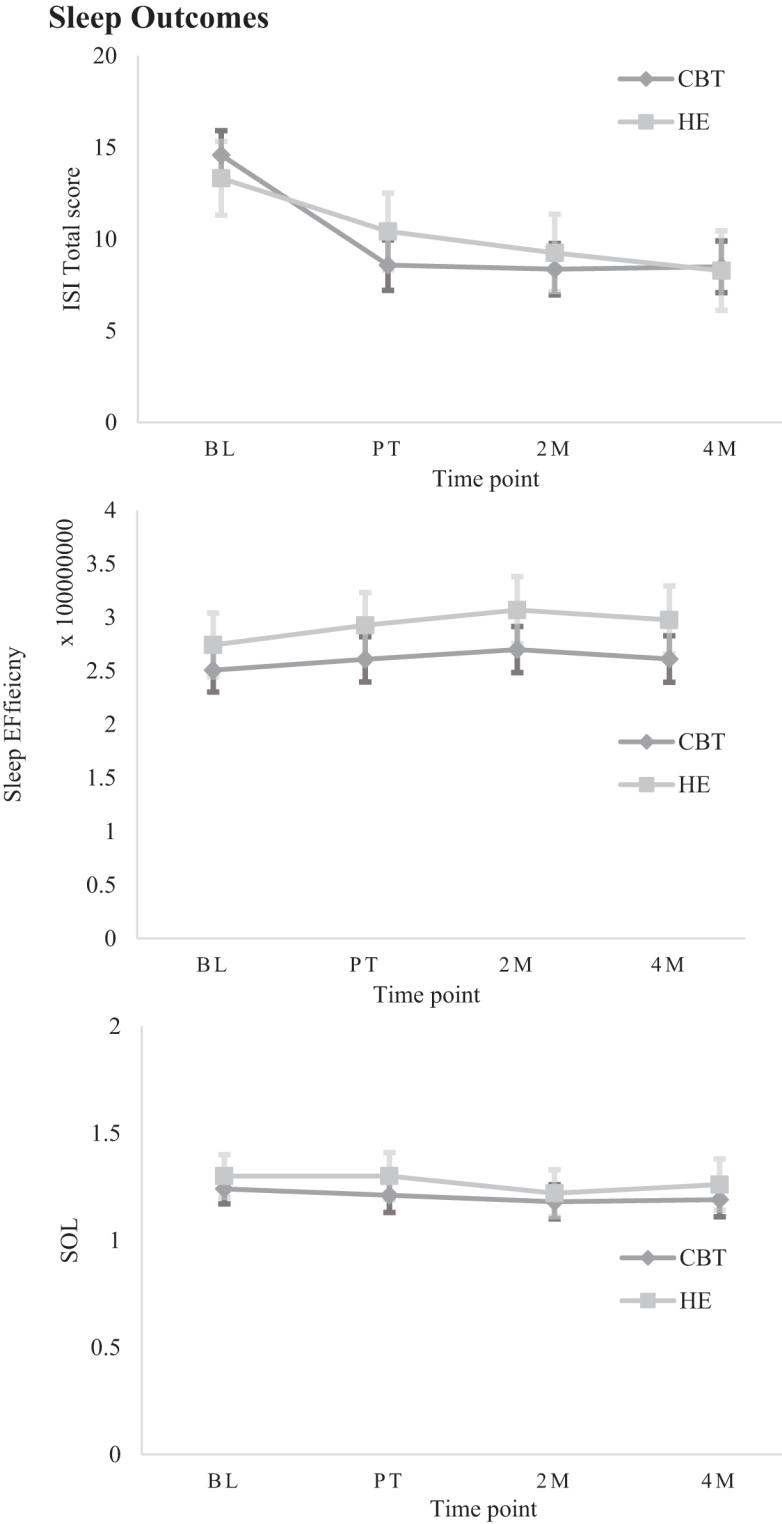
Estimated marginal means (± SE) on secondary measures for each group at each time point, grouped by category: Sleep.

**Fig. 4:**
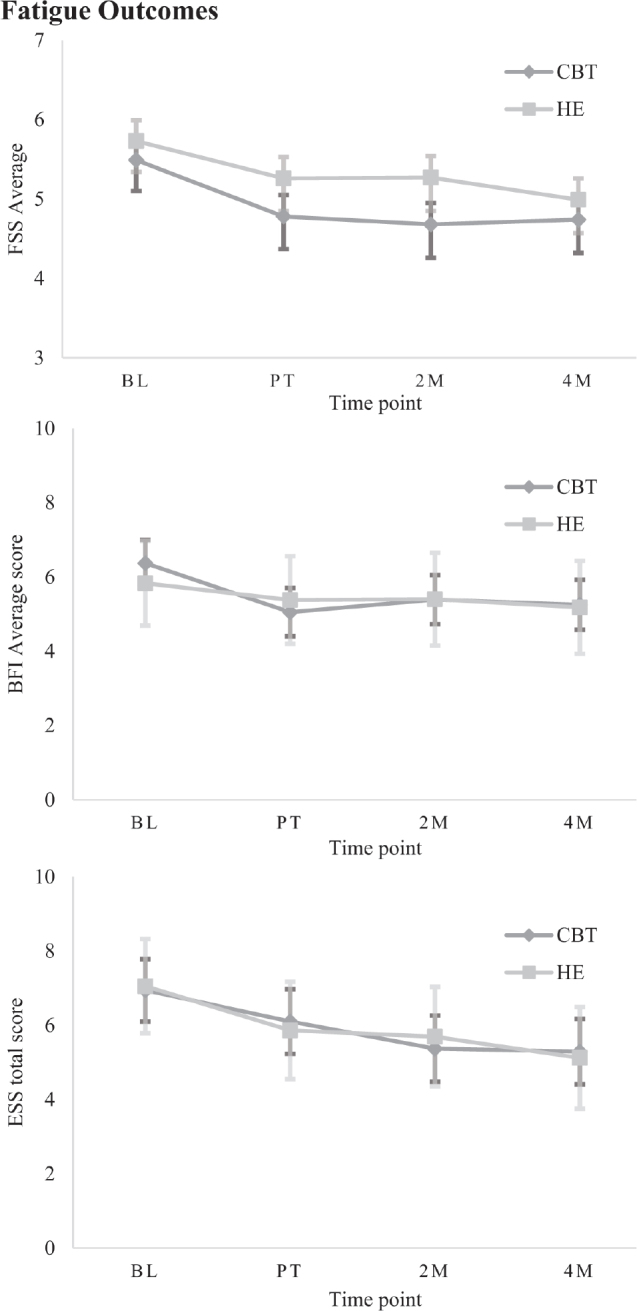
Estimated marginal means (± SE) on secondary measures for each group at each time point, grouped by category: Fatigue.

**Fig. 5:**
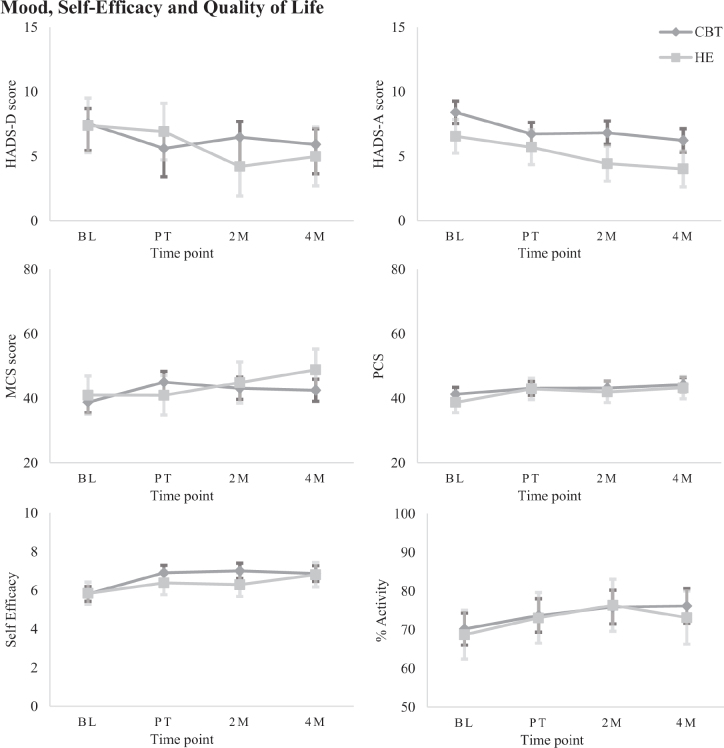
Estimated marginal means (± SE) on secondary measures for each group at each time point, grouped by category: Mood, Self-efficacy, and Quality of life.

*Sleep outcomes.* A significant Group*Time interaction was observed on the PSQI with a between-group difference immediately post-treatment, with CBT-SF improving significantly more than HE (*β* = –1.50, *p* < 0.001, 95% CI –2.35 to –0.64, see Tables SIII and SIV). There were no further between-groups differences at subsequent time points. Within each group, CBT-SF maintained significant improvements over time relative to baseline; with medium to large effect sizes (Cohen’s *d* = 0.69–0.80; Fig. 2), and HE had within-group improvements relative to baseline at 2 and 4 months post-treatment, with medium effect size (*d* = 0.54–0.74). These results were consistent in separate TBI and stroke groups, with the exception of the HE group showing only significant within-group gains at 4 months post-treatment and not at 2 months, for both injury types (see Tables SV and SVI). There was a significant Group*Time interaction on the ISI (*β* = –2.11, *p* = 0.005, 95% CI –3.58 to –0.65), where CBT-SF improved significantly more than HE immediately after treatment, with large effect size (*d* = 0.95). There were no further between-groups differences at subsequent time points (see Figs 3–5 for graphical representations of all secondary outcome measures). Both groups showed significant within-group gains relative to baseline scores immediately post-treatment and over time, with large effect for CBT-SF (*d* = 0.97–1.00), and moderate-to-large effect for HE (*d* = 0.43–0.81).

On actigraphy measures, there were separate main effects of Group (*β* = 32203606.27, *p* = 0.041, 95% CI 1671546.72–62735665.82) and Time (*β* = 13968911.53, *p* = 0.006, 95% CI 3930101.89–24007721.17) for Sleep Efficiency; however, after correcting for inflated *p*-values, no significant between or within-group differences were evident. No significant main effects or interactions were observed for SOL.

*Fatigue outcomes.* There was a significant Group*Time interaction effect on the FSS (*β* = –0.29, *p* = 0.047, 95% CI –0.58 to –0.01). While there were no significant between-groups differences at any post-treatment time points, there was a significant within-group reduction in fatigue for CBT-SF immediately after treatment and over time relative to baseline (*d* = 0.70–0.77). The HE group, by contrast, showed significant within-group gains only at 4 months post-treatment (*d* = 0.78). When examining TBI and stroke cohorts separately, the TBI group had no significant interaction effect. However, the same within-group changes were observed as in the whole sample, again with no between-group differences (*β* = –0.26, *p* = 0.383, 95% CI –0.74–0.28). In the stroke group, a significant Group*Time interaction revealed no between-group differences. However, CBT-SF improved significantly after treatment and maintained this over time, while there were no significant improvements after HE (*β* = –0.35, *p* = 0.048, 95% CI –0.70 to –0.01). On the BFI, there was a main effect of Time (*β* = 0.55, *p* = 0.013, 95% CI –0.98 to –0.12), with no between-groups differences but significant within-group improvements after CBT-SF treatment and over time (moderate effect, *d* = 0.63–0.75).

On the ESS, a main effect of Time identified no between-group differences. However, there was a significant within-group improvement after CBT-SF at 2- and 4-month follow-ups relative to baseline (*β* = –1.30, *p* < 0.001, 95% CI –1.76–0.85), with small effect size (*d* = 0.36–0.38). There was a significant small within-group improvement from baseline after HE at the 4-month follow-up (*d* = 0.46).

*Mood, self-efficacy, and quality of life.* There was a significant Group*Time interaction on the HADS-D (*β* = 2.22, *p* = 0.003, 95% CI 0.77–3.66). Simple effects analysis revealed no significant between-groups differences over time; however, CBT-SF showed within-group gains relative to baseline after treatment and at 4-month follow-up (*d* = 0.41–0.48), and HE improved at 2-month follow-up (*d* = 0.52). On the HADS-A, separate main effects of Group and Time were identified (*β* = –1.87, *p* = 0.011, 95% CI –3.29 to –0.45; *β* = –1.71, *p* < 0.001, 95% CI –2.16 to –1.27). There were no significant between-group differences but CBT-SF showed small-to-moderate within-group improvements across all time points compared with baseline (*d* = 0.41–0.55), and HE showed moderate gains at the 2-month and 4-month follow-ups (*d* = 0.67–0.77).

On the self-efficacy scale, there was a significant Group*Time interaction (*β* = 0.60, *p* = 0.012, 95% CI 0.13–1.07). While there were no significant between-groups differences, CBT-SF showed moderate within-group improvements immediately post-treatment and over time (*d* = 0.56–0.64), and HE made significant within-group gains at 4 months post-therapy only, with moderate effect size (*d* = 0.72).

A Group*Time interaction on the SF36v2 MCS (*β* = 5.41, *p* = 0.012, 95% CI 1.28–9.54), found no significant between-groups differences but CBT-SF showed small within-group gains post-therapy and at 2-month follow-up (*d* = 0.41–0.50). HE showed moderate within-group improvements at 4-month follow-up only (*d* = 0.65). On the SF36v2 PCS, there was a significant main effect of Time (*β* = 2.42, *p* < 0.001, 95% CI 1.28–3.56), with no between-groups differences but CBT-SF improved significantly at 4 months post-treatment relative to baseline (*d* = 0.33), and HE improved with small to moderate gains after treatment and at 4-month follow-up (*d* = 0.38–0.56).

When looking at time spent in productive activity, there was a main effect of Time (*β* = 4.09, *p* < 0.001, 95% CI 1.71–6.47). Simple effects did not identify between-groups differences, but within-group changes were present for CBT-SF only, improving significantly at 2- and 4-month follow-ups relative to baseline (*d* = 0.20–0.30).

### Clinically significant change over time on the primary outcome

Immediately post-treatment, 44.2% of participants (*n* = 38) who completed CBT-SF achieved clinically meaningful change on the PSQI, vs 7 participants (17.5%) in the HE group. At 2- and 4-month follow-ups, 32 (37.2%) and 34 (39.5%) participants respectively maintained clinically significant improvements in the CBT-SF group, as compared with 10 (25%) and 13 (32.5%) participants in the HE group.

## DISCUSSION

This study investigated the efficacy of CBT-SF compared with HE in treating sleep disturbance and fatigue in individuals with stroke and TBI. The hypothesis that those receiving CBT-SF would report significantly larger improvements in sleep quality compared with HE and maintain those gains over time was only partially supported. There were no significant group differences in sleep quality at follow-up time points. However, there were significantly larger improvements in CBT-SF compared with HE immediately after treatment. Positively, CBT-SF maintained sleep quality gains over time. Our additional hypothesis that those receiving CBT-SF would report greater improvements in fatigue, depression, anxiety, health-related quality of life, self-efficacy, actigraphy, and time spent in productive activity compared with HE was also partially supported, as there were no significant differences between groups over time, but significant changes within the CBT-SF group individually. Additionally, the CBT-SF group showed immediate and consistent gains across both FSS and BFI measures, whereas those receiving HE experienced delayed improvement on a single measure of fatigue only (FSS). CBT-SF also resulted in more rapid secondary improvements in self-efficacy, mood, and mental health-related quality of life based on within-group changes. Lastly, there were no significant changes on actigraphy variables, but significant increases in time spent in productive activity for the CBT-SF group only.

To our knowledge, this is the largest trial of this nature to date, and the first to evaluate an adapted CBT treatment for sleep and/or fatigue in an ABI population with a powered sample in relation to another intervention. The results extend the findings from our pilot trials (12, 13, 15), and those of previous uncontrolled pilot trials (11, 40). While both CBT-SF and HE interventions showed significant reductions in sleep and fatigue symptoms by 4 months post-treatment, the adapted CBT-SF programme produced larger benefits in sleep immediately after treatment, and appeared to be a more efficient means of reducing sleep and fatigue symptoms concurrently. Changes in fatigue were also more consistently observed across subjective measures of fatigue following CBT-SF. This suggests that the unique and tailored strategies offered in CBT-SF appear to be the primary driver of rapid symptom improvement in our sample, rather than non-specific therapy effects, in addition to the more generalized behaviour change strategies of CBT, such as goal setting and homework.

Participants who received HE, also delivered by a therapist over 8 weeks, showed a more gradual reduction in sleep disturbance and fatigue over a 4-month period following intervention. They also showed improvements in mood. Whilst the lack of rapid improvement during the intervention phase suggests this was not due to the therapist contact, it may have reflected a more gradual impact of lifestyle changes and associated changes in mood. The improvements in physical quality of life as measured by the SF36v2 PCS post-HE supports this contention. The benefits of health-related education and lifestyle modification on quality of life and risk factor management after ABI have been demonstrated previously (41). Therefore, treatment of sleep and fatigue disturbance after ABI should arguably include components of this health education.

Concurrent with improvements in sleep disturbance and fatigue, the CBT-SF treatment resulted in significant improvements in self-efficacy, mental health-related quality of life, and time spent in productive activity. Improvements in self-efficacy and quality of life were also observed after HE, but at a delayed time point, consistent with primary outcomes. This finding suggests that CBT for sleep and fatigue problems not only improves the primary symptoms, but also increases confidence in managing symptoms and capacity to engage in meaningful activities more rapidly than HE. This highlights the enduring impact of the CBT-SF intervention techniques and strategies, and their ability to produce lasting and holistic benefits.

There were no significant changes in objective measures of sleep, despite moderate-to-large improvements on subjective measures. As established by Mitchell et al. (42), changes in insomnia following CBT treatments are more robustly corroborated by subjective than objective measures, and associations between actigraphy and self-report measures may be less sensitive in TBI populations (43). While more accurate measures of sleep, such as polysomnography, could potentially identify objective sleep changes, these are not requisites for participants to experience positive and long-lasting changes in their sleep.

Due to travel restrictions associated with COVID-19 lockdowns as well as wide geographical distribution within Australia, a large proportion of participants completed their intervention sessions via videoconferencing. Positively, mode of treatment delivery was not a significant covariate contributing to any subjective outcome measures, and there were no significant differences in the proportion of participants in telehealth vs in-person modes between treatment groups. Past studies that have investigated online, telephone, and video interventions for treating sleep or fatigue problems in TBI and stroke populations have demonstrated feasibility and efficacy in reducing symptom severity (11, 44), although the present intervention appears to be one of the more intensive with regard to treatment length and duration. Encouragingly, 71% of participants who completed the treatment via telehealth stated that they would prefer telehealth or had no preference between telehealth and in-person modes. There was frequent feedback that videoconferencing was less taxing on fatigue, more flexible, and that rapport could be easily established via video. This corroborates growing evidence supporting telehealth approaches to facilitate greater access and availability of evidence-based treatments such as CBT-SF for individuals with ABI.

Strengths of our study include large sample size, high-quality RCT design, high treatment fidelity, and rigorous blinding of post-treatment assessors. However, limitations must be considered. While having an active control group assists in understanding the therapeutic mechanisms of symptom improvement, the lack of waitlist controls means we could not rule out the possibility of natural improvements in sleep or fatigue over time. Of note is that our previous pilot trials (12, 13) showed CBT-SF was superior to a waitlist control, and therefore the current trial aimed to establish the impact of specific CBT-SF components. Second, a large number of participants screened were not included in the study, largely due to ineligibility. Although having strict inclusion criteria is one of the strengths of an RCT, and while the treatment was administered to some individuals with significant cognitive impairments with appropriate adaptations, it inevitably results in decreased generalizability to broader ABI populations, including individuals who have comorbid severe psychiatric or neurological conditions, do not speak English, or have cognitive impairments precluding engagement in psychological therapy. Now that efficacy has been established, future studies could expand inclusion criteria and reach more individuals with ABI in the community. Third, we combined TBI and stroke cohorts in our study, and while there were minor differences between sleep and fatigue outcomes within each injury category, these were not considered to be sufficient to warrant separation within the main analysis, particularly given injury type was not a significant covariate for any subjective outcomes. However, given that our pilot analysis of predictors of treatment outcome found differing trajectories of fatigue between TBI and stroke (45), secondary analyses will be conducted and reported in a future paper. Fourth, participants were excluded if they had untreated symptomatic sleep apnoea, and were required to complete medical follow-up if they screened in the high-risk category. While it could be presumed that other sleep disorders (e.g., periodic limb movement disorder) would be identified during this process, given participants were not formally screened for other sleep disorders we cannot rule out that this may have impacted sleep and fatigue symptoms. Lastly, as stated above, our findings are based solely on subjective outcomes due to a lack of significant change observed on objective actigraphy measures, and we cannot rule out that the reliability of self-report diaries may also have been impacted by cognitive impairments. While we are confident that CBT-SF has produced a positive outcome with respect to sleep and fatigue symptoms in our sample, further investigation via use of polysomnography may assist in establishing whether objective sleep changes are present following CBT-SF.

Ultimately, this trial suggests that while both CBT-SF and HE may be beneficial in reducing sleep disturbance and fatigue after TBI and stroke, CBT-SF results in larger improvements in sleep immediately after treatment, as well as more rapid and consistent reductions in fatigue over time compared with HE. Improvements after CBT-SF were also more consistent across all measures of sleep and fatigue, whereas these were delayed and somewhat variable after HE. HE is likely to provide a foundation on which to build sleep and fatigue interventions; however, the unique and tailored strategies of CBT-SF appear more beneficial, particularly in the short term. Based on these findings we have greater confidence in the benefits of CBT-SF and the importance of addressing sleep disturbance and fatigue concurrently. The intervention has been manualized and we will embark on implementing the treatment into clinical practice, aiming to reach a more diverse group of individuals with ABI, to alleviate their sleep and fatigue problems and improve their quality of life.

## Supplementary Material


