# Supplementary material for: RANDOMIZED CONTROLLED TRIAL OF COGNITIVE BEHAVIOURAL THERAPY VERSUS HEALTH EDUCATION FOR SLEEP DISTURBANCE AND FATIGUE FOLLOWING STROKE AND TRAUMATIC BRAIN INJURY
Source: J Rehabil Med. 2025 Jan 3;57:41302. doi: 10.2340/jrm.v57.41302 (PMC11681142; doi:10.2340/jrm.v57.41302)
Supplement: Supplementary file 1 [file JRM-57-41302-s1.pdf]

Supplementary material has been published as submitted. It has not been copyedited, or typeset by Journal of Rehabilitation Medicine

Table SI. *Baseline demographic, cognitive and psychological characteristics by treatment condition for traumatic brain injury cohort (TBI)*

	CBT-SF (*n*=36) M (SD) or %	HE (*n*=15) M (SD) or %	Range
Demographics			
Age at study entry	42.00 (14.97)	42.51 (14.77)	19 – 71
Sex (% male)	56%	47%	-
Years of Education	13.44 (1.83)	12.79 (1.53)§	10– 17
Mode (%telehealth)	61%	73%	-
Cognitive characteristics			
NART IQ	108.47 (7.03)†	109.90 (9.28)§	89 – 121
CVLT-II T-score (trials 1-5)	45.46 (11.71)§	44.15 (12.81)‡	23 – 69
Psychological measures			
Ho-NOS ABI score	7.69 (4.63)	7.79 (3.62)§	0 – 19
Baseline HADS-D	6.61 (3.47)	5.43 (4.20)§	0 – 17
Baseline HADS-A	8.67 (4.85)	7.64 (4.13)§	0 – 20
Baseline BPI	4.63 (1.36)	2.90 (1.09)	0 – 7

Note. BPI, Brief Pain Inventory; CBT-SF, cognitive behavioural therapy for sleep disturbance and fatigue; CVLT-II, California Verbal Learning Test – second edition; HADS-A, Hospital Anxiety and Depression Scale – Anxiety subscale; HADS-D, Hospital Anxiety and Depression Scale – Depression subscale; HE, health education; Ho-NOS-ABI, Health of the Nation Outcome Scale – Acquired Brain Injury; M, mean; NART IQ, National Adult Reading Test Intelligence Quotient; SD, standard deviation

†Missing data from between four to eight participants

‡Missing data from two participants

§Missing data from one participant

Table SII. *Baseline demographic, cognitive and psychological characteristics by treatment condition for stroke cohort*

	CBT-SF (*n*=50)<br>M (SD) or %	HE (*n*=25)<br>M (SD) or %	Range
Demographics			
Age at study entry	50.71 (13.35)	53.65 (11.46)	23 – 71
Sex (% male)	52%	52%	-
Years of Education	13.66 (1.91)	13.84 (2.61)	9 – 18
Mode (%telehealth)	86%	80%	-
Cognitive characteristics			
NART IQ	111.85 (5.93)‡	112.71 (5.87)§	98 – 121
CVLT-II T-score (trials 1-5)	50.59 (12.75)§	49.40 (11.81)	12 – 77
Psychological measures			
Ho-NOS ABI score	7.04 (3.61)	6.38 (4.64)§	0 – 19
Baseline HADS-D	6.52 (3.63)	6.42 (3.45)§	0 – 17
Baseline HADS-A	8.22 (3.47)	5.92 (3.76)§	0 – 16
Baseline BPI	3.95 (1.73)	3.48 (2.25)	1 – 7

Note. BPI, Brief Pain Inventory; CBT-SF, cognitive behavioural therapy for sleep disturbance and fatigue; CVLT-II, California Verbal Learning Test – second edition; HADS-A, Hospital Anxiety and Depression Scale – Anxiety subscale; HADS-D, Hospital Anxiety and Depression Scale – Depression subscale; HE, health education; Ho-NOS-ABI, Health of the Nation Outcome Scale – Acquired Brain Injury; M, mean; NART IQ, National Adult Reading Test Intelligence Quotient; SD, standard deviation

‡Missing data from between four participants

§Missing data from one participant

Table SIII. *Mixed multilevel modelling results for primary and secondary outcome measures*

Measure	Coeff	95% CI	*p*-value
PSQI			
Group	0.38	-1.07 – 1.82	.610
Time***	0.79	0.36 – 1.22	<.001
Baseline HADS-A*[†]	0.19	0.03 – 0.35	.025
Group x Time***	-1.50	-2.35 – -0.64	<.001
ISI			
Group	0.31	-1.83 – 2.45	.776
Time***	2.02	1.28 – 2.75	<.001
Group x Time**	-2.11	-3.58 – -0.65	.005
FSS			
Group	0.36	-0.04 – 0.81	.075
Time**	0.24	0.10 – 0.38	.001
Baseline HADS-A**	0.07	0.03 – 0.12	.002
Group x Time*	-0.29	-0.58 – -0.01	.047
BFI			
Group	-0.07	-1.12 – 1.08	.912
Time*	-0.55	-0.98 – -0.12	.013
Baseline HADS-A	0.02	-0.10 – 0.14	.764
Baseline BPI*	0.35	0.06 – 0.65	.023
Group x Time	0.25	-0.62 – 1.11	.578
ESS			
Group	0.01	-1.37 – 1.38	.993
Time***	-1.30	-1.76 – 0.85	<.001
Group x Time	-0.07	-0.98 – 0.84	.885
Sleep efficiency[‡]			
Group*	3.22E+07	1.67E+06 - 6.27E+07	.041
Time**	1.40E+07	3.93E+06 - 2.40E+07	.006
Mode	3.07E+07	-2.73E+06 - 6.41E+07	.075
Injury Type**	4.31E+07	1.39E+07 - 7.22E+07	.005
Group x Time	9.89E+06	-1.02E+07 - 3.00E+07	.334
Sleep onset latency[§]			
Group	0.07	-0.04 - 0.18	.224
Time	-0.04	-0.09 – 0.01	.083
Baseline HADS-A	0.02	0.00 – 0.03	.012

Group x Time	-0.01	-0.10 – 0.08	.883
HADS-D			
Group	-0.51	-2.68 – 1.66	.646
Time	0.17	-0.56 – 0.89	.653
Baseline HADS-A	0.15	-0.07 – 0.38	.194
Baseline BPI*	0.62	0.07 – 1.18	.033
Group x Time**	2.22	0.77 – 3.66	.003
HADS-A			
Group*	-1.87	-3.29 – -0.45	.011
Time***	-1.71	-2.16 – -1.27	<.001
Group x Time	-0.54	-1.43 – 0.35	.238
SF36 Mental Component Summary			
Group	1.55	-4.49 – 7.60	.617
Time	-0.71	-2.78 – 1.36	.502
Baseline HADS-A**	-1.08	-1.71 – -0.45	.002
Baseline BPI	-1.55	-3.10 – 0.01	.057
Group x Time*	5.41	1.28 – 9.54	.012
SF36 Physical Component Summary			
Group	-1.12	-4.36 – 2.22	.491
Time***	2.42	1.28 – 3.56	<.001
Group x Time	0.85	-1.43 – 3.13	.464
Self-efficacy			
Group	-0.32	-0.92 – 0.28	.302
Time**	-0.31	-0.55 – -0.08	.009
Baseline HADS-A***	-0.13	-0.19 – -0.06	<.001
Group x Time*	0.60	0.13 – 1.07	.012
% Productive Activity			
Group	-1.16	-7.88 – 5.56	.736
Time***	4.09	1.71 – 6.47	<.001
Group x Time	-0.77	-5.52 – 3.99	.752

PSQI, Pittsburgh Sleep Quality Index; ISI, Insomnia Severity Index; FSS, Fatigue Severity Scale; BFI, Brief Fatigue Inventory; ESS, Epworth Sleepiness Scale; HADS-D, Hospital Anxiety and Depression Scale – Depression subscale; HADS-A, Hospital Anxiety and Depression Scale – Anxiety subscale; SF36, Short Form Health Survey; BPI, Brief Pain Inventory

***Significant at $p<.001$

**Significant at $p<.01$

*Significant at $p<.05$

†Covariates on which groups differed at baseline were included in analysis if significantly impacting the analysis outcome

‡BOXCOX transformation applied

§Logarithm transformation applied

Table SIV. *Means, standard deviations and effect sizes for each group across time*

	Group	Timepoint	Mean	SD	Cohen's *d*
PSQI	CBT	Baseline	9.80	4.26	
		Post-therapy	6.41	3.66	0.80
		2-months	6.85	3.71	0.69
		4-months	6.67	3.57	0.73
	HE	Baseline	8.89	4.99	
		Post-therapy	7.59	4.22	0.26
		2-months	6.19	3.89	0.54
		4-months	5.18	3.08	0.74
ISI	CBT	Baseline	14.59	6.30	
		Post-therapy	8.62	5.63	0.95
		2-months	8.27	5.76	1.00
		4-months	8.46	5.51	0.97
	HE	Baseline	13.32	7.71	
		Post-therapy	9.97	7.10	0.43
		2-months	8.26	6.67	0.66
		4-months	7.07	6.42	0.81
FSS	CBT	Baseline	5.54	1.04	
		Post-therapy	4.81	1.33	0.70
		2-months	4.74	1.34	0.77
		4-months	4.79	1.37	0.72
	HE	Baseline	5.63	1.05	
		Post-therapy	5.12	1.29	0.49
		2-months	5.11	1.30	0.50
		4-months	4.81	1.38	0.78
BFI	CBT	Baseline	5.72	1.74	
		Post-therapy	4.42	2.24	0.75
		2-months	4.62	2.16	0.63
		4-months	4.61	2.31	0.64
	HE	Baseline	5.44	2.01	
		Post-therapy	4.88	2.17	0.28
		2-months	4.69	2.30	0.37
		4-months	4.44	2.07	0.50
ESS	CBT	Baseline	6.94	4.23	
		Post-therapy	6.14	4.04	0.19
		2-months	5.41	3.50	0.36

		4-months	5.34	3.67	0.38
	HE	Baseline	7.05	4.44	
		Post-therapy	6.00	3.80	0.24
		2-months	5.81	4.09	0.28
		4-months	5.00	3.94	0.46
Sleep Efficiency	CBT	Baseline	82.02	6.45	
		Post-therapy	82.77	6.5	-0.12
		2-months	84.05	5.62	-0.31
		4-months	82.7	7.07	-0.11
	HE	Baseline	83.81	6.74	
		Post-therapy	85.74	4.48	-0.29
		2-months	86.06	4.84	-0.33
		4-months	86.06	3.43	-0.33
Sleep Onset Latency	CBT	Baseline	22.67	18.94	
		Post-therapy	20.69	15.93	0.10
		2-months	20.6	21.79	0.11
		4-months	22.17	22.35	0.03
	HE	Baseline	24.52	19.73	
		Post-therapy	21.03	11.3	0.18
		2-months	19.66	14.93	0.25
		4-months	19.02	10.31	0.28
HADS-A	CBT	Baseline	8.41	4.08	
		Post-therapy	6.62	4.22	0.44
		2-months	6.72	4.68	0.41
		4-months	6.16	4.23	0.55
	HE	Baseline	6.55	3.94	
		Post-therapy	5.42	3.89	0.29
		2-months	3.90	3.56	0.67
		4-months	3.50	2.86	0.77
HADS-D	CBT	Baseline	6.56	3.54	
		Post-therapy	4.87	3.23	0.48
		2-months	5.48	4.13	0.31
		4-months	5.10	3.86	0.41
	HE	Baseline	6.05	3.72	
		Post-therapy	5.12	3.67	0.25
		2-months	4.10	3.43	0.52
		4-months	3.18	2.98	0.77

SF-36 Mental Component Summary	CBT	Baseline	39.88	11.57	
		Post-therapy	45.65	11.03	-0.50
		2-months	44.65	13.09	-0.41
		4-months	44.47	12.50	-0.40
	HE	Baseline	43.98	10.29	
		Post-therapy	43.99	11.19	0.00
		2-months	47.88	10.79	-0.38
		4-months	50.64	7.98	-0.65
SF-36 Physical Component Summary	CBT	Baseline	41.28	9.87	
		Post-therapy	43.18	10.01	-0.19
		2-months	43.15	10.24	-0.19
		4-months	44.49	10.35	-0.33
	HE	Baseline	38.74	9.85	
		Post-therapy	42.85	8.81	-0.42
		2-months	42.44	9.60	-0.38
		4-months	44.28	9.57	-0.56
Self-Efficacy	CBT	Baseline	5.72	1.88	
		Post-therapy	6.78	2.01	-0.56
		2-months	6.92	1.80	-0.64
		4-months	6.82	1.83	-0.59
	HE	HE	5.99	1.54	
		Post-therapy	6.56	2.03	-0.37
		2-months	6.56	1.73	-0.37
		4-months	7.10	1.81	-0.72
% Productive Activity	CBT	Baseline	70.28	19.43	
		Post-therapy	74.21	19.13	-0.20
		2-months	76.09	17.66	-0.30
		4-months	75.56	19.96	-0.27
	HE	HE	68.69	18.17	
		Post-therapy	72.78	16.87	-0.23
		2-months	76.16	21.03	-0.41
		4-months	75.37	19.59	-0.37

CBT, Cognitive Behavioural Therapy; HE, Health Education; PSQI, Pittsburgh Sleep Quality Index; ISI, Insomnia Severity Index; FSS, Fatigue Severity Scale; BFI, Brief Fatigue Inventory; ESS, Epworth Sleepiness Scale; HADS-D, Hospital Anxiety and Depression Scale – Depression subscale; HADS-A, Hospital Anxiety and Depression Scale – Anxiety subscale; SF36, Short Form Health Survey

Table SV. *Means and standard deviations on the PSQI and FSS for separate TBI and stroke groups*

			TBI		Stroke	
	Group	Timepoint	Mean	SD	Mean	SD
PSQI	CBT	Baseline	10.36	4.55	9.40	4.04
		Post-therapy	6.30	3.91	6.48	3.53
		2-months	6.37	4.06	7.14	3.49
		4-months	6.31	3.13	6.89	3.13
	HE	Baseline	10.64	4.52	7.88	5.05
		Post-therapy	9.17	4.02	6.65	4.15
		2-months	7.18	3.16	5.65	4.22
		4-months	5.90	3.03	4.78	3.12
FSS	CBT	Baseline	5.30	1.22	5.72	0.86
		Post-therapy	4.62	1.46	4.95	1.24
		2-months	4.50	1.36	4.88	1.33
		4-months	4.70	1.46	4.85	1.33
	HE	Baseline	5.56	0.99	5.67	1.10
		Post-therapy	4.82	1.42	5.30	1.22
		2-months	4.80	1.10	5.29	1.39
		4-months	4.52	1.20	4.98	1.48

TBI, traumatic brain injury; CBT, Cognitive Behavioural Therapy; HE, Health Education; PSQI, Pittsburgh Sleep Quality Index; FSS, Fatigue Severity Scale

Table SVI. *Mixed multilevel modelling results for PSQI and FSS within separate TBI and stroke groups*

	TBI			Stroke		
Measure	Coeff	95% CI	*p*-value	Coeff	95% CI	*p*-value
PSQI						
Group	1.78	-0.50 – 4.05	.133	-0.81	-2.56 – 0.95	.371
Time	-2.51	-3.23 – -1.78	*<.001*	-1.59	-2.13 – -1.05	*<.001*
Baseline HADS-A	0.27	0.05 – 0.49	*.020*	-	-	-
Group x Time	-2.07	-3.49 – -0.65	*.005*	-1.12	-2.19 – -0.05	*.041*
FSS						
Group	0.26	-0.42 – 0.94	.451	0.25	-0.26 – 0.77	.340
Time	-0.57	-0.83 – -0.32	*<.001*	0.20	0.03 – 0.38	*.023*
Baseline HADS-A	0.11	0.04 – 0.17	*.003*	-	-	-
Group x Time	-0.23	-0.74 – -0.28	.383	-0.35	-0.70 – -0.01	*.048*

TBI, traumatic brain injury; PSQI, Pittsburgh Sleep Quality Index; FSS, Fatigue Severity Scale; HADS-A, Hospital Anxiety and Depression Scale – Anxiety

Significant at p<.05
